# Antibiotics Knowledge, Attitudes and Behaviours among the Population Living in Cyprus

**DOI:** 10.3390/antibiotics12050897

**Published:** 2023-05-12

**Authors:** Mark J. M. Sullman, Timo J. Lajunen, Buket Baddal, Menelaos Apostolou

**Affiliations:** 1 Department of Social Sciences, School of Humanities and Social Sciences, University of Nicosia, Nicosia CY-1700, Cyprus; sullman.m@unic.ac.cy (M.J.M.S.); apostolou.m@unic.ac.cy (M.A.); 2 Department of Psychology, Norwegian University of Science and Technology, NO-7491 Trondheim, Norway; 3 Department of Medical Microbiology and Clinical Microbiology, Faculty of Medicine, Near East University, Nicosia 99138, Cyprus; buket.baddal@neu.edu.tr

**Keywords:** antibiotics, resistance, attitude, knowledge, Republic of Cyprus, Turkish Republic of Northern Cyprus

## Abstract

This study investigated the knowledge, attitudes and behaviours towards antibiotics among the general public living in the Republic of Cyprus (RoC) and the Turkish Republic of Northern Cyprus (TRNC) by using an online questionnaire. Differences were examined using independent samples *t*-tests, chi-square tests, Mann–Whitney U tests and Spearman’s rho. In total, 519 individuals completed the survey (RoC = 267, TRNC = 252), with an average age of 32.7, and 52.2% were female. Most citizens correctly identified paracetamol (TRNC = 93.7%, RoC = 53.9%) and ibuprofen (TRNC = 70.2%, RoC = 47.6%) as non-antibiotic medications. A substantial proportion thought antibiotics could treat viral infections, such as a cold (TRNC = 16.3%, RoC = 40.8%) or the flu (TRNC = 21.4%, RoC = 50.4%). Most participants understood that bacteria can become resistant to antibiotics (TRNC = 71.4%, RoC = 64.4%), that unnecessary use can lead to drug ineffectiveness (TRNC = 86.1%, RoC = 72.3%) and that they should always complete the course of antibiotics (TRNC = 85.7%, RoC = 64.0%). Positive attitudes towards antibiotics correlated negatively with knowledge in both samples, indicating that the more people know, the less positive their attitudes towards their use. The RoC appears to have tighter controls of over-the-counter (OTC) sales of antibiotics than TRNC. This study reveals that different communities can have varying levels of knowledge, attitudes and perceptions about antibiotic use. Tighter enforcement of the OTC regulations, educational efforts and media campaigns are needed for enhancing prudent antibiotic use on the island.

## 1. Introduction

According to the European Centre for Disease Control, in 2020, the Republic of Cyprus (RoC) consumed 28.9 defined daily doses (DDD) per 1000 inhabitants, which is the highest in the European Union (EU) and considerably higher than the statistic of 8.5 found in the Netherlands [1]. In 2012, the Ministry of Health published a National Strategic Plan to combat antimicrobial resistance in the Republic of Cyprus. This plan included several different types of measures (e.g., awareness campaigns, controls on the use of critically important antimicrobials for humans and clear recommendations on the prudent use of antimicrobials), but antibiotic use remains the highest in the EU.

The island of Cyprus has been divided in two since 1974 based on ethnic identity, with the Greek Cypriots living in the south (Republic of Cyprus) and the Turkish Cypriots living in the northern part of the island (Turkish Republic of Northern Cyprus—TRNC). The north of the island is not covered by the EU statistics or those from the World Health Organization (WHO), and so very little is known about the situation there. Although there are no readily available official statistics on antibiotic use in the TRNC, a 2014 study found that 87% of the 300 patients surveyed had purchased antibiotics without a prescription at least once in their life [2]. Furthermore, a 2014 survey of half of the pharmacies in the TRNC found that 97.6% of pharmacists sold antibiotics to patients without a prescription [3]. It is important to note that these two studies were conducted before April 2016, when selling antibiotics without a prescription became illegal in the TRNC.

The misuse of antibiotics is a global problem that represents one of the largest threats to human health and food security [4]. The WHO has stated that the unnecessary and inappropriate use of antibiotics is one of the main factors underpinning the development of antimicrobial resistance (AMR). AMR not only compromises the effectiveness of life-saving medical interventions, such as intensive care, cancer treatment and organ transplantation, but it is also one of the largest threats to food security.

Interventions aimed at increasing the level of knowledge regarding the appropriate use of antibiotics among patients and healthcare professionals, as well as improving communication between the two, are important steps towards combating antibiotic resistance [5]. However, in order to be able to generate appropriate interventions for both parties, it is essential to have a thorough understanding of both patients’ and healthcare professionals’ levels of knowledge, understanding and attitudes regarding antibiotic use and antibiotic resistance [6]. There have been a number of studies investigating the attitudes and knowledge relating to antibiotic use in the general population [7,8,9,10,11,12,13], which have revealed substantial intercountry differences in antibiotic knowledge and practice. In addition, diverse health systems, healthcare practices and education related to the use of antibiotics indicate that research is required for a better understanding of the local contexts in each country or territory.

Two recent studies have investigated the knowledge, attitudes, and behaviours among those living in Cyprus, one in the TRNC [8] and the other in the Republic of Cyprus [9]. Ilktac et al. (2020) surveyed 701 adults living in the TRNC and found that 61.9% of the participants were either currently using antimicrobial therapy or had done so in the past 12 months. The majority (70%) had intermediate/high knowledge of antibiotic use, with almost two-thirds (66%) having heard about antibiotic resistance, and 96% of those had intermediate/high knowledge (64% and 32%, respectively). University graduates were more likely than those with only primary school level education to recognize the term antibiotic resistance. Almost a fifth (18%) of the participants reported having used antibiotics leftover by a family member or friend, and 27% stated that they stopped taking antibiotics when the symptoms disappeared. In terms of the disorders that antibiotics can cure, 72% correctly believed that antibiotics can treat bladder infections, while over a quarter (26.5%) thought that antibiotics could be used to treat viral infections (including human immunodeficiency virus—HIV), and over 22% thought they could treat a cold or the flu.

Michaelidou et al. (2020) surveyed 614 people living in the Republic of Cyprus and found that around one-third had used antibiotics in the last 12 months and up to 72.3% had heard about “antibiotic resistant bacteria”. Surprisingly, 70.7% did not understand how antibiotic resistance developed, but most agreed on the usefulness of the suggested actions to address antibiotic resistance. Among respondents, 87% stated that they knew they needed to take the full prescription of antibiotics, and 79.6% understood that it was not acceptable to use the same antibiotics taken from a family member or friend with the same symptoms. When participants were asked to select which medical conditions should be treated with antibiotics, bladder/UTI (81%), skin/wound infections (61.9%) and gonorrhea (23.1%) were correctly identified, but a common cold/flu (36.6%), sore throat (30.0%) and HIV/AIDS (6%) were incorrectly identified. As with the TRNC survey, those with higher education exhibited significantly higher knowledge levels. A better understanding of the knowledge and attitudes about antibiotic and antibiotic resistance in the general population of both parts of Cyprus may be helpful for identifying the problematic attitudes, beliefs and behaviours that exist in Cyprus. The purpose of the present study was to investigate the current level of knowledge about antibiotic use and antibiotic resistance among the general public in the two ethnic communities living on the island of Cyprus. This information could help guide future interventions to improve awareness and reduce unnecessary antibiotic use in both communities.

## 2. Results

### 2.1. Population Characteristics

The data included answers from 267 respondents from the Republic of Cyprus (RoC) and 252 respondents from the Turkish Republic of Northern Cyprus (TRNC). The sample characteristics are described in Table 1. The table shows that the sample from TRNC was somewhat older, included more females, included more married people, and had a higher education level than the sample from RoC. The respondents were also asked about smoking, keeping fit, being in good health and having a health professional background. The TRNC sample included fewer smokers, considered themselves to be more fit and included slightly more people with a health professional background than the RoC sample. There was no statistically significant difference in self-evaluated health. In general, both samples comprised highly educated people, of which 82% in the RoC and 91% in the TRNC had obtained a university degree.

### 2.2. Previous Experiences with Antibiotic Use

The respondents were asked about their previous experiences with antibiotic use (Table 2). Almost half of the respondents had used antibiotics in the last 12 months. Among the users, the most common was to have had only one course of antibiotics (Table 2). Respondents from TRNC were more likely to follow the doctor’s orders when using antibiotics than those from RoC. In general, the participants were observed to follow the doctor’s instructions well. When asked about requesting a prescription for antibiotics, although unnecessary, the RoC community reported having asked for an unnecessary course of antibiotics more often than those from TRNC did. It should be noted that asking for a prescription for antibiotics without reason was not common: the great majority of the RoC and TRNC residents indicated that they had never done so. Interestingly, the majority of the respondents from TRNC reported that it is extremely easy or easy to obtain antibiotics without a prescription, while the opposite results were found among the RoC respondents.

### 2.3. Recognition of Antibiotics

Table 3 shows the distribution of answers among the RoC and TRNC study participants and the Mann–Whitney tests for country comparisons. The most correctly recognized antibiotic was penicillin, with 53.2% in the RoC, and amoxicillin in the TRNC (72.2%). In general, the respondents from TRNC recognized medicines better than those from the RoC. The only medication with equal recognition rates in both samples was penicillin. This is not surprising considering the long history of penicillin use. More surprisingly, 22.5% of RoC residents thought that paracetamol was an antibiotic, while the same figure for the TRNC was 3.6%. In summary, the country comparisons (Mann–Whitney statistics) show that there were significant intercountry differences, and in most cases, the differences indicated better knowledge among TRNC residents than those living in the RoC. The knowledge about antibiotics, in terms of recognition rates, was worryingly low. Without the knowledge of which pharmaceuticals are antibiotics and which are not, patients cannot be expected to use the medications as prescribed.

### 2.4. Antibiotic Use and Effects

Table 4 shows the differences between the RoC and TRNC samples in terms of knowledge about antibiotic use and its effect. Statistical differences between the RoC and TRNC populations were found in 9 of the 13 statements. In every case where there were significant differences between samples, respondents from the TRNC showed better knowledge than those from the RoC. Interestingly, statements in which sample differences were not found were more related to side effects (diarrhea) or interactions with other medications (Table 4). The RoC community believed, more often than the TRNC community, that using antibiotics with alcohol or all kinds of food was harmless. In statements related to the effectiveness and use of antibiotics (items 1, 2, 3 and 4), TRNC respondents had better knowledge than the RoC respondents. The RoC respondents believed that antibiotics kill viruses and are effective against colds and flu more often than the TRNC respondents did. In the situation where 50.4% of respondents believe that antibiotics are effective against the flu and 40.8% believe that antibiotics are effective against colds, misuse of antibiotics can be expected to be very likely.

### 2.5. Resistance and the Prevention Measures

Participants were asked questions about the development of antibiotic resistance (13 questions, see Table 5). In general, most participants were aware of the risk of antimicrobial resistance (question 1; 64.4% in the RoC and 71.4% in the TRNC had a correct answer), the risk of unnecessary use of antibiotics (question 7; 86.1% in the TRNC and 72.3% in the RoC) and the need to complete a course of antibiotics, even if feeling better (85.7% in the TRNC and 64.0% in the RoC). The most common misunderstandings were related to viruses becoming resistant (question 1; 34.9% in the TRNC and 41.6% in the RoC answered “yes”). Hence, the majority of individuals were found to recognize the risks of using antibiotics too often or incorrectly, while the exact mechanisms of how antibiotic resistance develops were less well known.

Mann–Whitney U statistics showed that the two samples differed from each other in 6 of the 13 statements. In general, the TRNC participants were more knowledgeable about facts related to antibiotic resistance. Only in the responses to item 13 (“the more antibiotics we use in society, the higher the risk that resistance develops and spreads”), the RoC participants were more likely to answer ‘yes’ (51.3%) than those from the TRNC (46.8%).

### 2.6. Attitudes to Antibiotic Use

Attitudes towards antibiotics use were measured using ten statements, and the answers were recorded with a 5-point Likert scale from “strongly disagree” (1) to “strongly agree” (5). Table 6 presents the means (M) and standard deviations (SD) for both samples and the related pairwise comparisons (*t*-tests). Respondents from the TRNC agreed more often with statements 1, 3, 4 and 6 than the RoC respondents, while the RoC participants agreed more often with statements 2, 5 and 9. Items 1, 3 and 6 reflect a negative or strict attitude to antibiotic use, while items 2, 5 and 9 reflect a more relaxed attitude towards antibiotic use. Interestingly, TRNC residents scored higher on both items 3 and 4, even though they reflect somewhat opposite attitudes. It should be noted that among both RoC (t(251) = −10.80, *p* < 0.001) and TRNC (t(265) = −11.47, *p* < 0.001) samples, respondents found it more important that the GP does not prescribe an antibiotic when he/she thinks that they are not needed than prescribing them when the patient requests them. Based on group comparisons in attitude items, it can be concluded that TRNC citizens preferred a stricter control of antibiotic use than the RoC citizens.

## 3. Discussion

Public awareness of the causes and consequences of antibiotic resistance is critical for the mitigation of the inappropriate use of antibiotics. Therefore, the assessment of the awareness, knowledge and attitude regarding antibiotic use and antibiotic resistance represents an important tool for identifying areas within populations for the implementation of targeted interventions and initiatives for increasing public awareness. In the current study, the knowledge, attitudes and behaviour regarding antibiotic and antibiotic resistance were examined in the two different communities living on the island of Cyprus in order to determine similarities and differences between the communities.

The restriction of antibiotic dispensing without a prescription is among the most important measures against non-prudent antibiotic use in a given country. In EU member states, the sale of antibiotics without a prescription is prohibited by law. Nevertheless, an increase in the use of antibiotics without a prescription in Cyprus, Bulgaria, Croatia, Finland, Germany, Latvia, Lithuania and Poland was reported between 2013 and 2016, with the prevailing source being a pharmacy [14]. This implies that not enforcing the law on over-the-counter (OTC) sales is the main driver for the use of antibiotics without a prescription. In the current study, the vast majority of the TRNC respondents (83.7%) indicated that it is extremely easy or easy to obtain antibiotics without a prescription, while only 25.6% of the RoC participants were able to do so, indicating a significant difference in the antibiotic stewardship between the two communities. The rates of self-medication may also vary between different countries. In Italy, a study focusing on attitudes, knowledge and antibiotic use among the general public reported that a minority (32.7%) of the study participants had used antibiotics without a prescription, and only 22.7% were willing to take an antibiotic without a prescription. In a separate study, based on the Romanian population, 78.9% of the participants indicated that they would only consume antibiotics on doctor’s prescription, while a small percentage (10.3%) stated that they would take antibiotics if they felt sick, regardless of a doctor’s prescription [15]. In a 2019 survey of 1044 participants, performed face to face in Turkey, only 27.6% of the individuals had used antibiotics without a prescription [16], which was significantly lower than the 64.3% who reported self-medicating in the same city in 2014, two years prior to the banning of the antibiotics sales without a prescription [17]. Notably, a 2023 global meta-analysis of self-medication revealed that the mean incidence of self-medication was higher in Eastern Europe and Asian countries compared to other parts of the world [18].

In an effort to evaluate the knowledge level of those living in the two communities in Cyprus, we assessed their ability to recognize antibiotics from five given pharmaceuticals (tetracycline, penicillin, ibuprofen, paracetamol and amoxicillin). The study results showed that overall, TRNC citizens were better able to recognize the medications than citizens from RoC, and they were more able to recognize amoxicillin and penicillin as antibiotics. Among the RoC participants, 22.5% incorrectly identified paracetamol as an antibiotic, which was much lower (3.6%) for TRNC participants. These rates were lower than in low-income countries, such as Indonesia and Nepal, where 41.2% and 28.5% of the population identified paracetamol as an antibiotic, respectively [18,19,20]. In a 2020 study conducted among the general public in southern Italy, 89.5% of the citizens correctly categorized amoxicillin as an antibiotic, while only 12.9% thought paracetamol was an antibiotic [21]. Furthermore, a survey conducted in 2021 among the general population of Spain found that individuals less than 65 years old were better at differentiating between antibiotics and other types of medication than those in the over-65 age group [22]. Indeed, lack of public knowledge has been implicated as a driver of the unnecessary use of antibiotics among the general public in Europe [23].

As an individual or a parent, it is important to know against which microorganisms antibiotics are effective or not. Indeed, this knowledge level correlates with the rates of antibiotic resistance in a community. In the current study, RoC respondents were found to have a considerably lower level of knowledge regarding the effectiveness of antibiotics. Among the RoC respondents, 50.4% of them believed that antibiotics were effective against the flu, and 40.8% believed they were effective against colds. In such a situation, the misuse and overuse of antibiotics in the community is very likely. Similar persisting misconceptions regarding the effectiveness of antibiotics can exist in communities living in different geographical locations. A 2021 study in Turkey revealed that 37% of parents with a child under 18 years old believed that antibiotics could cure infections caused by viruses [24]. In France, only about half of the participants in the general public knew that antibiotics only target bacteria, regardless of whether they were parents with a child ≤6 years old or not [25]. A similar finding was observed among Italian parents, among which 33% declared that antibiotics were useful for treating viral infections [26]. In a Swedish population survey, this rate was lower, with only 13.4% of the participants incorrectly stating that antibiotics make people recover faster from a cold [11]. In addition, a study of 620 householders in Jordan indicated there was a poor understanding of antibiotic usage among the Jordanian public, as only 14.2% of the sample in West Amman and 2.9% in East Amman disagreed with the statement “antibiotics work on most coughs and colds”. The study also revealed that Jordanians perceived pharmacists as strong influencers in their decision, with 80.3% of the surveyed population having used antibiotics in the last year following pharmacists’ advice [27]. Interestingly, about 14% of the respondents in a survey of 516 participants from Bulgaria believed that they could treat SARS-CoV-2 infection with antibiotics [28].

The knowledge of the mechanisms of the development of antibiotic resistance can be a driver of better attitudes regarding antibiotic use. In general, most participants in both communities were aware of the risk of antimicrobial resistance (64.4% in the RoC and 71.4% in the TRNC having a correct answer), the risk of the unnecessary use of antibiotics (86.1% in the TRNC and 72.3% in the RoC) and the need of completing the full course of antibiotics, even if feeling better (85.7% in the TRNC and 64.0% in the RoC). These rates are higher than those in previous studies undertaken in different countries. For example, in a study by Pogurschi et al., the authors reported that the Romanian population was not disciplined enough when it comes to completing antibiotic treatments, as 29.2% of the respondents indicated they would stop the course of antibiotics if their symptoms improved [15]. In a cross-sectional study of 2406 Syrian respondents, a high proportion of the citizens (65.3%) were aware that antibiotic resistance will spread faster as a result of improper antibiotic usage [29]. Recently, in a survey of 15,526 Chinese antibiotic users, Yin et al. revealed that 53.3% of the respondents were non-adherent to antibiotic treatment, and the most commonly observed non-adherence behaviour was discontinuing antibiotics early (78.0%), followed by missing antibiotics (48.3%), decreasing antibiotic dosage (25.5%) and increasing antibiotic dosage (15.2%) [30]. Similarly, in a public survey performed during a mass gathering in India, 87% of the 1915 participants reported failing to comply with the prescribed course of antibiotics, and 88.5% had inappropriate responses for practice [31]. Misconceptions about antibiotic resistance were identified in our study, in which the most common misunderstandings were related to viruses becoming resistant (34.9% in the TRNC and 41.6% in the RoC answered “yes”). Misconceptions about antibiotic resistance were also identified in the general public of India, with 52% of the respondents stating that humans can become resistant to antibiotics [32].

In the current study, both RoC and TRNC respondents found it more important that the GP does not prescribe an antibiotic when he/she thinks that they are not needed than prescribing them when the patient requests them. Based on group comparisons in attitude items, our study revealed that TRNC citizens preferred a stricter control of antibiotic use than RoC citizens did. In a similar study in Lebanon, the authors reported that almost half of the respondents (48.4%) expected their doctors to prescribe antibiotics to treat a common cold, with 15.4% of these respondents having asked their physicians to prescribe antibiotics when they suffered from common cold symptoms [33]. In a meta-analysis conducted in Australia and Sweden, overall, 28% of respondents indicated that they would go to see another doctor when their (first) doctor did not prescribe or provide antibiotics. In the same meta-analysis, which included four different studies from Australia and the United Kingdom, on average, 27% of the individuals reported that they would expect and want to receive antibiotics for a viral infection, such as the cold and flu [34]. In contrast, during a face-to-face survey of randomly selected households across England, 84.0% of the participants stated that they would be pleased if their general practitioner (GP) said they did not need antibiotics, and only 21% would challenge the GP’s decision not to prescribe antibiotics [35].

Notably, the differences in self-medication with antibiotics between Cyprus and other countries observed in this study are due to the availability of OTC sales of antibiotics on the island, in particular in the TRNC region. In many countries, including Cyprus, OTC sales of antibiotics are prohibited by law; yet, this does not necessarily mean that antibiotics are not sold OTC in those countries. Strategies to enforce regulations prohibiting OTC sales of antibiotics, such as retention of the prescriptions by pharmacies, government inspections, educational activities for healthcare workers and media campaigns for the general public, are among the approaches that should be implemented in both parts of Cyprus. Nevertheless, law enforcement requires resources for effective medicine registration systems, a good capacity for inspections and a legal system to impose penalties for breaches of regulations, none of which are in place in the TRNC. The results revealed that the recognition of antibiotics and their effectiveness, as well as the mechanisms of antibiotic resistance development, should be improved via educational efforts targeting both adults and children. Furthermore, a multifaceted approach should be conducted that combines education for school children, campaigns for the adult population and antibiotic stewardship programs for medical professionals. Appropriate curricula should be developed for medical and nonmedical undergraduate students about general medicines, the virulence of microorganisms, mechanism of antibiotic resistance and prudent antibiotic prescribing.

The results of our study should be interpreted in the context of several limitations. The majority of the respondents from the RoC and TRNC were higher education graduates, with either a university/college degree ≤ 4 years or a university/college degree > 4 years. This could indicate that the accessibility of the survey was lower in certain geographical areas, such as rural areas, which may have introduced sampling bias. The study was based on a self-administered online survey in which the results were based on the honest feedback of the participants. This limitation was minimized by the anonymous nature of the study, as no personal identifiers were collected.

This study also has several strengths. To the best of our knowledge, this is the first study to investigate and compare the knowledge and attitudes regarding antibiotics and antibiotic resistance among citizens of the RoC and TRNC. Hence, it provides important information and insights into the similarities and differences between the two populations living on this divided island. This study also contributes to our understanding of the issues in each of the two populations regarding antibiotic knowledge and use, which can be used by government agencies to implement strategies to address them, with a potential impact on healthcare in both Cypriot communities.

## 4. Materials and Methods

### 4.1. Study Design and Population

The study design was a cross-sectional survey because the aim was to describe different aspects of antibiotic use, knowledge and attitudes in the Republic of Cyprus (RoC) and in the Turkish Republic of Northern Cyprus (TRNC). The populations chosen for the study were people living or studying in the RoC or TRNC.

### 4.2. Participants and Data Collection

Sample size estimation with G*Power 3.1 application [36] (effect size 0.5, power 0.95) indicated that the minimum sample size for sample comparisons was 184. Participants were obtained via advertising on social media (e.g., Facebook) and snowball sampling. They were asked to complete an online questionnaire hosted by Google Forms. Participants received a link to the questionnaire, which contained information on the background to the study, objectives and voluntary nature of participation, as well as declarations of anonymity and the confidentiality of all data.

### 4.3. Informed Consent and Ethics Information

Informed consent was obtained from all participants, and the Social Science Ethics Research Board (SSERB) at the University of Nicosia (SSERB 00137) approved the study.

### 4.4. Questionnaire

The questionnaire consisted of six sections. Section 1 asked participants to report their history of antibiotic use over the last 12 months [7,11,13]. This included whether they had taken antibiotics in the past 12 months and, if they had, how many courses they had taken (Once, 2–5 times or More than 5 times). This section also asked whether they had followed their doctor’s advice, if they had ever taken antibiotics without a doctor’s prescription and how easy it would be to obtain antibiotics without a prescription.

In Section 2, participants were presented with five types of drugs (tetracycline, penicillin, ibuprofen, paracetamol and amoxicillin) and were asked whether each was an antibiotic and to record their answers using one of three options: No (1), Don’t Know (2) or Yes (3).

Section 3 consisted of two parts; the first measured participant’s knowledge about antibiotics (e.g., antibiotics kill viruses) [13], and the second asked questions about antibiotic resistance (e.g., bacteria may become resistant towards antibiotics) [11,13]. Both the knowledge about antibiotics and resistance were measured with 13 state-ments, and were answered using a 5 point Likert scale (1 = Strongly agree to 5 = Strongly disagree). In Section 4, participants were asked to answer 10 questions concerning their personal attitudes towards antibiotics (e.g., I want to use antibiotics only if it is necessary), which were answered on a five-point Likert scale (strongly agree to strongly disagree). The questions for this section were sourced from earlier studies [11,13,37]. The final section asked participants to report their sex, age, marital status, whether they smoked, two questions about their self-perceived general health, household income, nationality and native language.

### 4.5. Statistical Analyses

All analyses were performed using IBM SPSS for Windows, version 27 (IBM, Japan). The knowledge, attitudes and practices of participants from the different communities were compared using independent samples *t*-tests, chi-square tests and Mann–Whitney U statistics. A *p* value of 0.05 or less was considered to be significant.

## 5. Conclusions

In conclusion, the current study reveals that despite the less-controlled OTC sales and consumption of antibiotics in the TRNC, the citizens were more educated and knowledgeable about antibiotics, their effects, the risks of antibiotic resistance and its prevention compared to RoC citizens. This further highlights that within the same island, different communities can have varying levels of knowledge, attitude and perceptions regarding antibiotic use and antibiotic resistance. As TRNC and RoC citizens can freely visit both parts and interact with each other, the implementation of interventions should be targeted at both communities in order to ensure the appropriate control of antibiotic resistance on the island. These interventions should primarily include enforcing the existing legislation prohibiting over-the-counter (OTC) sales and multifaceted educational campaigns both at the community level and for community pharmacists in order to increase awareness and improve antibiotic use.

## Figures and Tables

**Table 1:** Sample characteristics.

	RoC		TRNC		
Variable	M or N	SD or %	M or N	SD or %	*T*-Test or χ^2^
Age	26.2	10.4	39.5	13.6	*p* < 0.001
Sex					*p* < 0.001
Female	109	40.8	162	64.3	
Male	153	57.3	86	34.1	
Missing	5	1.9	4	1.6	
“What is your marital status?”					*p* < 0.001
Married/Cohabiting	44	16.5	160	63.5	
Single	130	48.7	54	21.4	
In relationship	85	31.8	29	11.5	
Other	8	3.0	9	3.6	
“What is your highest educational qualification?”					*p* < 0.001
Primary school/lower secondary school	3	1.1	3	1.2	
Upper secondary school/high school	42	15.7	20	7.9	
University/College ≤ 4 years	123	46.1	86	34.1	
University/College > 4 years	97	36.3	143	56.7	
Missing	2	0.7	0	0.0	
“Do you have a health professional background?”					*p* < 0.05
Yes	31	11.6	48	19	
No	216	80.9	204	81	
Missing	20	7.5	0	0.0	
“Do you smoke?”					*p* < 0.01
Yes	72	27.0	48	19.0	
No	155	58.1	184	73.0	
Sometimes	38	14.2	20	7.9	
Missing	2	0.7	0	0.0	
“I keep myself physically fit”					*p* < 0.05
Strongly agree	59	22.1	39	15.5	
Agree	112	41.9	106	42.1	
Neither agree nor disagree	68	25.5	74	29.4	
Disagree	24	9.0	24	9.5	
Strongly disagree	4	1.5	9	3.6	
“I am in good health”					*p* = NS
Strongly agree	55	20.6	42	16.7	
Agree	140	52.4	151	59.9	
Neither agree nor disagree	48	18.0	45	17.9	
Disagree	22	8.2	10	4.0	
Strongly disagree	2	0.7	4	1.6	

Notes: M denotes mean; N denotes count; SD denotes standard deviation.

**Table 2:** Antibiotic use and perceived availability in TRNC and RoC.

	RoC		TRNC		
Variable	M or N	SD or %	M or N	SD or %	χ^2^
“Have you taken antibiotics in the last 12 months?”					*p* < 0.05
Yes	132	49.4	111	44.0	
No	115	43.1	138	54.8	
Don’t know	20	7.5	3	1.2	
“How many times have you taken a course of antibiotics during the past 12 months?”					*p* = NS
Never	131	49.1	137	54.4	
Once	83	31.1	74	29.4	
2–5 times	46	17.2	33	13.1	
More than 5 times	7	2.6	8	3.2	
“The last time you used antibiotics, did you follow the doctor’s instructions on dosage and length of treatment?”					*p* < 0.05
Yes	217	81.3	229	90.9	
No	41	15.4	13	5.2	
Don’t know	9	3.4	7	2.8	
Missing	0	0.0	3	1.2	
“Have you ever asked a doctor for antibiotics, although the doctor deemed it unnecessary?”					*p* < 0.05
Yes, and received for myself	26	9.7	12	4.8	
Yes, and received for my child	9	3.4	2	0.8	
Yes, but did not receive	13	4.9	3	1.2	
No	219	82	235	93.3	
“How easy to obtain antibiotics without a prescription?”					*p* < 0.05
Extremely easy	17	6.4	124	49.2	
Easy	51	19.1	87	34.5	
Neither easy nor difficult	117	43.8	34	13.5	
Difficult	63	23.6	6	2.4	
Extremely difficult	18	6.7	1	0.4	
Missing	1	0.4	0		

Notes: M denotes mean; N denotes count; SD denotes standard deviation.

**Table 3:** Recognition of antibiotics and a community comparison (Mann–Whitney U statistics).

	RoC			TRNC			Mann–Whitney U	Difference
Variable	No %	Don’t Know %	Yes %	No %	Don’t Know %	Yes %		
Tetracycline	7.9	57.7	34.5	3.6	55.2	41.3	*p* < 0.05	TRNC > RoC
Penicillin	13.9	33.0	53.2	19.8	15.1	65.1	*p* = NS	-
Ibuprofen	47.6	33.7	16.9	70.2	19.4	10.3	*p* < 0.001	RoC > TRNC
Paracetamol	53.9	21.7	22.5	93.7	2.8	3.6	*p* < 0.001	RoC > TRNC
Amoxicillin	9.4	46.8	43.4	2.4	25.4	72.2	*p* < 0.001	TRNC > RoC

**Table 4:** Knowledge about antibiotic use and the effects among RoC and TRNC populations.

	RoC			TRNC			Mann–Whitney U	Country Difference
Variable	No %	Don’t Know %	Yes %	No %	Don’t Know %	Yes %		
1. Antibiotics kill viruses.	41.9	16.9	41.2	66.7	10.3	23.0	*p* < 0.001	RoC > TRNC
2. Antibiotics are effective against bacteria.	10.2	18.0	71.8	6.7	10.7	82.5	*p* < 0.01	TRNC > RoC
3. Antibiotics are effective against colds.	41.9	17.2	40.8	75.0	8.7	16.3	*p* < 0.001	RoC > TRNC
4. Antibiotics are effective against flu.	33.8	15.8	50.4	69.8	8.7	21.4	*p* < 0.001	RoC > TRNC
5. Penicillin is another word for antibiotics.	36.7	41.6	21.7	53.6	25.0	21.4	*p* < 0.01	RoC > TRNC
6. One can get well from a bacterial infection without the use of antibiotics.	17.6	36.0	46.4	25.4	20.2	54.4	*p* = NS	-
7. Antibiotics may also kill beneficial bacteria that we normally carry on our skin or in our stomach/intestines.	8.6	31.8	59.6	6.3	15.1	78.6	*p* < 0.001	TRNC RoC
8. Antibiotics treatment often cause side effects like diarrhoea.	11.2	47.2	41.6	17.9	27.0	55.2	*p* = NS	-
9. One side effect from antibiotics treatment is vaginal fungus infection in women.	6.4	54.3	39.3	8.3	28.6	63.1	*p* < 0.001	TRNC > RoC
10. Antibiotics may influence the effect of other medications.	4.5	28.8	66.7	5.6	25.4	69.0	*p* = NS	-
11. Other medications may influence the effect of antibiotics.	5.2	27.3	67.4	9.5	25.4	65.1	*p* = NS	-
12. For some antibiotics combination with alcohol can be dangerous.	4.1	16.5	79.4	3.2	7.1	89.7	*p* < 0.01	TRNC > RoC
13. Antibiotics can be used together with all kinds of food.	31.8	41.2	27.0	49.2	30.2	20.6	*p* < 0.001	RoC > TRNC

**Table 5:** Knowledge about antibiotic resistance and its effects among RoC and TRNC participants.

	RoC			TRNC			Mann–Whitney U	Community Difference
Variable	No %	Don’t Know %	Yes %	No %	Don’t Know %	Yes %		
1. Bacteria may become resistant to antibiotics.	7.5	27.3	64.4	9.5	19.0	71.4	*p* = NS	-
2. Viruses may become resistant to antibiotics.	24.3	33.3	41.6	38.5	26.6	34.9	*p* < 0.01	RoC > TRNC
3. Humans may become resistant to antibiotics.	9.0	27.3	62.9	15.1	15.1	69.8	*p* = NS	-
4. One can be a “carrier” of resistant bacteria, which means to have resistant bacteria in the body without being ill.	5.6	44.6	48.7	10.3	37.3	52.4	*p* = NS	-
5. Infections by resistant bacteria is increasing in Cyprus.	4.9	67.0	27.3	4.0	51.6	44.4	*p* < 0.001	TRNC > RoC
6. Resistant bacteria is a problem in Cyprus hospitals.	5.2	64.4	29.6	6.3	51.6	42.1	*p* < 0.05	TRNC > RoC
7. Unnecessary use of antibiotics can make them less effective.	7.1	18.7	72.3	5.2	8.7	86.1	*p* < 0.001	TRNC > RoC
8. If you feel well halfway through the treatment that the doctor ordered, you can stop the antibiotics treatment.	64.0	19.5	15.4	85.7	4.8	9.5	*p* < 0.001	RoC > TRNC
9. Cypriots can help to prevent antibiotics resistance.	9.7	45.7	43.8	11.5	26.6	61.9	*p* < 0.001	TRNC > RoC
10. Frequent use of antibiotics on animals can reduce the effectiveness of antibiotics in humans.	22.8	56.2	20.2	25.8	47.6	26.6	*p* = NS	-
11. Antibiotic resistance can spread from person to person.	40.1	41.9	17.2	41.3	35.7	23.0	*p* = NS	-
12. Antibiotic resistance can spread from animals to humans	39.3	47.2	12.7	38.1	42.1	19.8	*p* = NS	-
13. The more antibiotics we use in society, the higher the risk that resistance develops and spreads.	10.5	37.5	51.3	23.4	29.8	46.8	*p* < 0.05	RoC > TRNC

**Table 6:** Attitudes to antibiotic use among the RoC and TRNC populations.

	RoC		TRNC		
	M	SD	M	SD	t
1. I want to use antibiotics only if it is necessary.	4.60	0.63	4.86	0.44	*p* < 0.001
2. I want to use antibiotics if it makes me get well sooner.	3.85	1.07	3.65	1.23	*p* = NS
3. The doctor should give me antibiotics when I think I need it.	2.68	1.36	3.44	1.53	*p* < 0.001
4. The doctor should not give me antibiotics when he/she thinks I do not need it.	4.03	1.20	4.60	0.92	*p* < 0.001
5. Leftover antibiotics can be saved for personal use in the future or given to someone else.	2.55	1.19	2.31	1.25	*p* < 0.05
6. Leftover antibiotics should be taken back to the pharmacy.	2.63	1.03	2.83	1.23	*p* < 0.05
7. It is good that one needs a prescription to acquire antibiotics from pharmacies in Cyprus.	4.15	0.97	4.13	1.15	*p* = NS
8. It is good to be able to buy antibiotics online, without having to see a doctor.	1.91	1.09	2.02	1.18	*p* = NS
9. It is good to be able to acquire antibiotics from relatives or acquaintances, without having to be examined by a doctor.	2.07	1.10	1.84	1.12	*p* < 0.05
10. It is good that one can buy antibiotics without a prescription in pharmacies in Cyprus.	2.07	1.19	2.04	1.19	*p* = NS

## Data Availability

The datasets generated and analyzed during the current study are not publicly available due to the fact that the datasets include multiple extensive files with a large number of variables, but they are available from the corresponding author upon reasonable request.
